# Warm sitz bath as an adjunctive therapy for distal ureteral calculi ≤5 mm: a prospective study

**DOI:** 10.3389/fmed.2026.1881597

**Published:** 2026-07-01

**Authors:** Li-diao Li, Jing-jing Lou, Ze-peng Wu, Xiang Hu, Hai-ou Lv, Yu-han He

**Affiliations:** Department of Urology, Yongkang First People's Hospital Affiliated to Hangzhou Medical College, Jinhua, Zhejiang Province, China

**Keywords:** analgesics, baths, pain measurement, treatment outcome, ureteral calculi, urolithiasis

## Abstract

**Objective:**

To evaluate the efficacy of warm sitz bath (WSB) as an adjunctive conservative therapy for patients with distal ureteral calculi (DUC) ≤5 mm.

**Methods:**

A total of 131 eligible patients with DUC ≤5 mm were prospectively randomized into the WSB group or the control group. Patients in the WSB group received standardized WSB (40–42 °C, 15–20 min, once daily), whereas those in the control group received no expulsive intervention. All patients were required to complete a 4-week follow-up after enrollment.

**Results:**

Of the 131 patients, 121 completed follow-up and were included in the final analysis, including 59 patients in the WSB group and 62 patients in the control group. Baseline characteristics were comparable between the two groups (all *P* > 0.05). The WSB group demonstrated significantly shorter stone expulsion time compared with the control group (9.3 ± 2.9 days vs. 14.3 ± 4.2 days, *P* < 0.001). Stone expulsion rates (SER) at week 1 (40.7% vs. 21.0%, *P* = 0.019) and week 2 (81.4% vs. 61.3%, *P* = 0.015) were significantly higher in the WSB group, whereas no significant difference was observed in the 4-week stone expulsion rate (93.2% vs. 87.1%, *P* = 0.260). The WSB group also showed significantly fewer daily pain episodes (0.8 ± 0.3 vs. 1.3 ± 0.4, *P* < 0.001), lower post-treatment VAS scores (3.1 ± 1.0 vs. 4.2 ± 1.7, *P* < 0.001), and lower analgesic requirements (4.1 ± 1.5 vs. 8.4 ± 3.1, *P* < 0.001). No significant differences in adverse events were observed between the two groups.

**Conclusion:**

WSB may shorten the expulsion time of DUC ≤5 mm and reduce pain burden and analgesic requirements; however, it does not significantly improve the overall 4-week SER.

## Introduction

Urolithiasis is one of the most common diseases encountered in urological practice and represents an increasing public health burden worldwide (1). Recent epidemiological evidence indicates that the global burden of urinary stone disease continues to rise, with approximately 106 million incident cases reported in 2021 (1). In the United States, approximately one in ten individuals is affected by urinary stone disease, imposing a considerable economic burden on both patients and the healthcare system (2, 3).

Owing to anatomical characteristics of the ureter, the distal ureter represents the most frequent site of stone impaction and retention (4). According to the European Association of Urology (EAU) Guidelines, distal ureteral calculi (DUC) < 5 mm have an approximately 89% chance of spontaneous passage (5). Alpha-blockers have been reported to increase the expulsion rate of ureteral stones larger than 5 mm; however, they offer minimal benefit for stones smaller than 5 mm (5–10). Given the high likelihood of spontaneous passage, current guidelines recommend watchful waiting rather than medical expulsive therapy (MET) for stones smaller than 5 mm (5, 11, 12). However, observation is not always an uneventful or comfortable process. During expectant management, patients may experience recurrent episodes of renal colic, repeated analgesic use, and, in some cases, urinary tract infection. In more severe situations, particularly when obstruction is complicated by infection, the clinical course may progress to urosepsis (5, 11). Therefore, although spontaneous passage is common in this population, strategies that may facilitate earlier stone passage and reduce symptom burden remain clinically relevant.

Warm sitz baths (WSB) are a simple, inexpensive, non-pharmacological intervention that has been used in clinical practice mainly for symptom relief, muscle relaxation, and alleviation of local discomfort (13–15). In urology, they have been used chiefly as an adjunctive measure for chronic prostatitis/chronic pelvic pain syndrome and for selected postoperative pelvic or perineal discomfort (13–15). Nevertheless, their role in the management of ureteral stones has not been adequately investigated.

Therefore, the present study was designed to evaluate the potential value of warm sitz baths in patients with DUC ≤5 mm, specifically whether this intervention could accelerate stone passage and improve pain-related outcomes.

## Patients and methods

**Figure 1:**
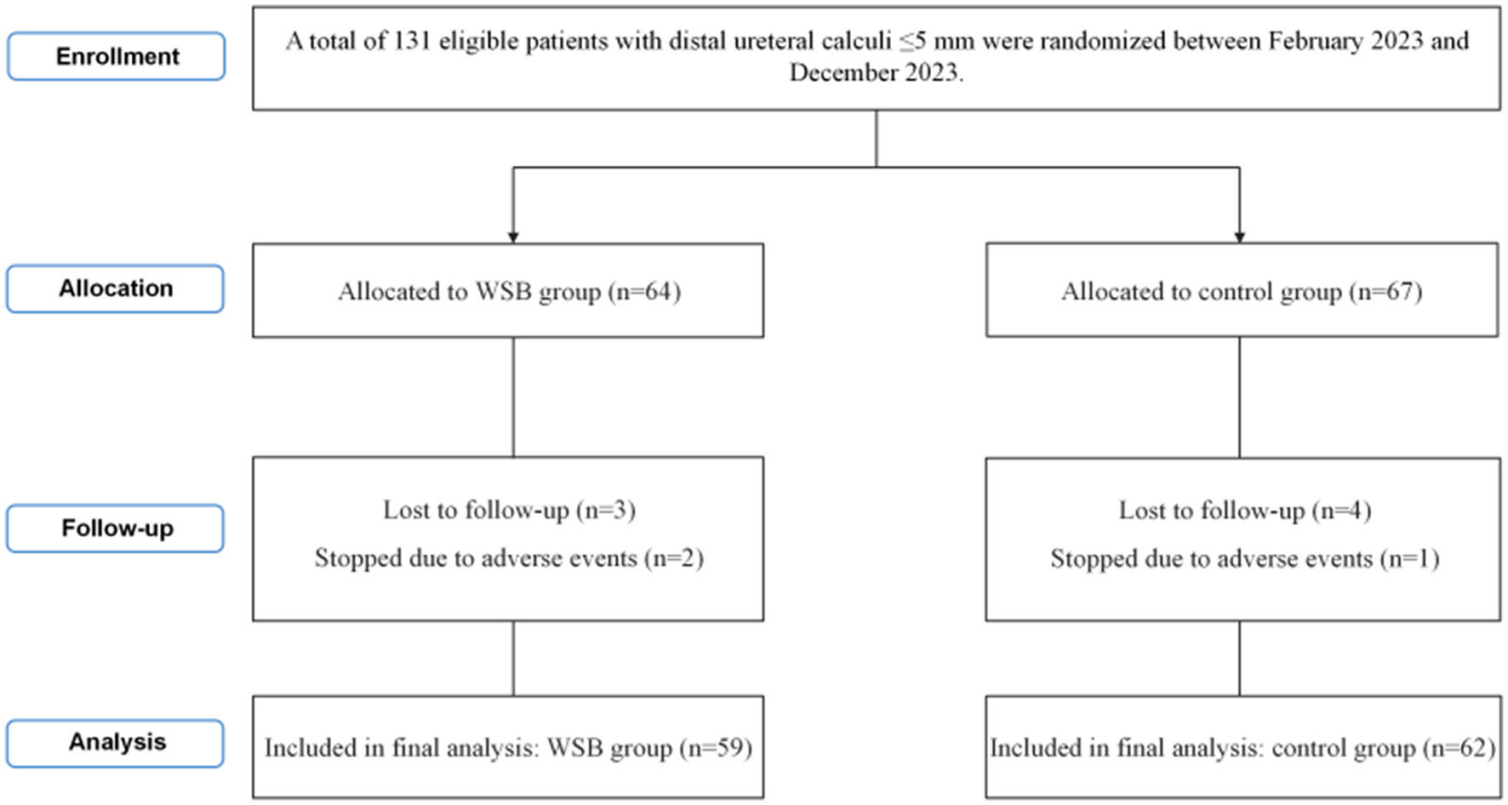
Flowchart of patient enrollment, randomization, follow-up, and analysis.

From February 2023 to December 2023, patients presenting to our hospital with renal colic who were diagnosed with DUC ( ≤5 mm), fulfilled the following inclusion criteria, and provided informed consent were enrolled in this study. The inclusion criteria were as follows: (1) age 18–65 years; (2) single DUC ≤5 mm; (3) first presentation to the hospital with renal colic; (4) normal renal function. The exclusion criteria were as follows: (1) urinary tract infection or urosepsis; (2) proximal or mid-ureteral stone location; (3) prior ipsilateral ureteral surgery; (4) pregnancy; (5) inability to perform WSB. Patients with urinary tract infection were treated with appropriate antibiotics and enrolled only after clinical resolution and normalization of infection-related laboratory parameters. Stone diagnosis was established by abdominal ultrasonography, kidney-ureter-bladder (KUB) radiography, or non-contrast computed tomography (CT). Stone size was defined as the maximum diameter measured on the imaging modality used. During follow-up, ultrasonography was performed as the first-line imaging modality to minimize radiation exposure; plain X-ray was added when ultrasonographic findings were inconclusive, and CT was reserved as the last-line modality when necessary. This study complied with the 1,964 Declaration of Helsinki and received approval from the Ethics Committee of the Yongkang First People's Hospital Affiliated to Hangzhou Medical College (YKSDYRMYYEC2023-KT-HS-012-01). All participants provided written informed consent. The flow of patient enrollment, randomization, follow-up, and analysis is illustrated in Figure 1.

**Figure 2:**
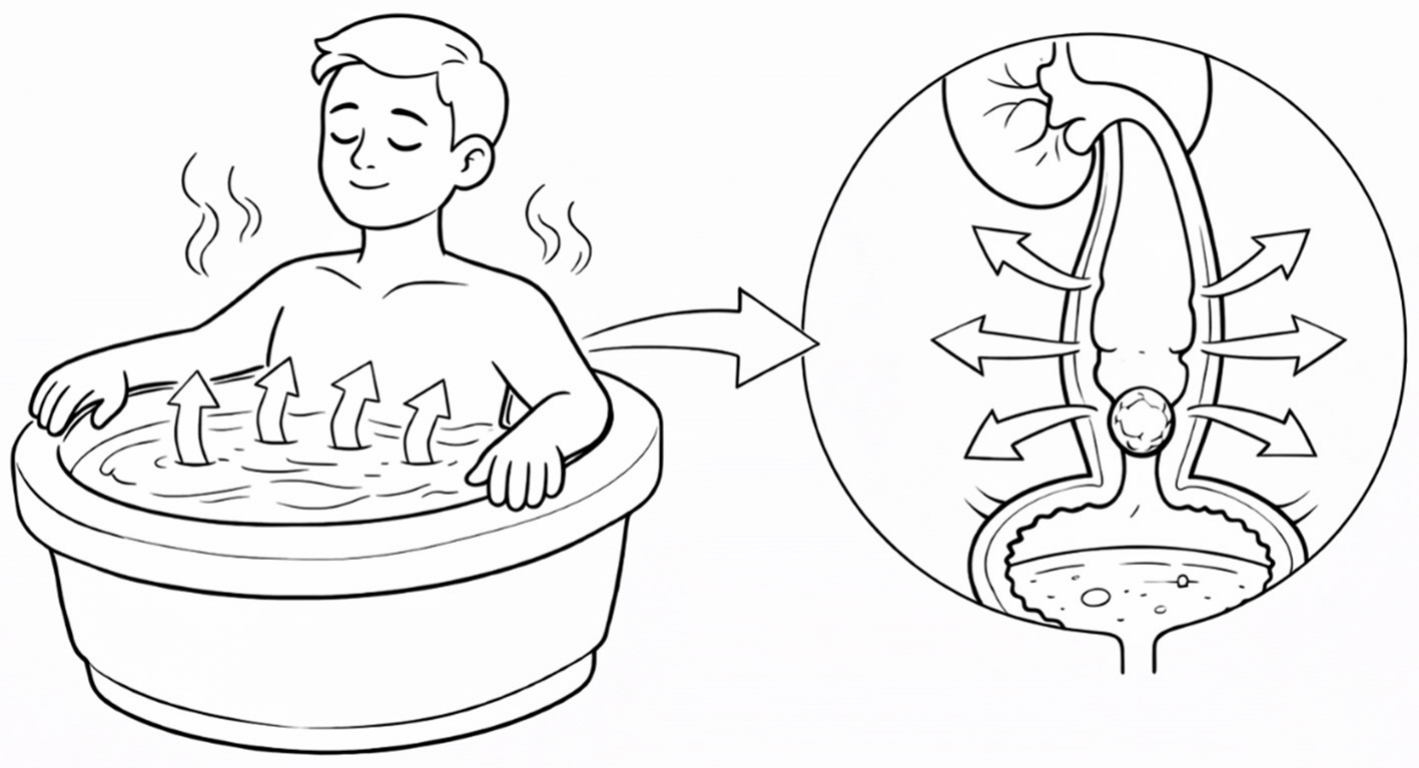
Schematic illustration of the WSB procedure and the hypothesized mechanism by which it may facilitate DUC passage.

Eligible participants were randomly assigned in a 1:1 ratio to either the WSB group or the control group. The randomization sequence was generated using a computer-generated random number table before patient enrollment. To ensure allocation concealment, group assignments were placed in sequentially numbered, opaque, sealed envelopes, which were opened only after a participant had been confirmed eligible and had provided written informed consent. Due to the nature of the warm sitz bath intervention, participant blinding was not feasible. Patients in the WSB group were instructed to undergo a sitz bath once daily in the evening (water temperature, 40–42 °C, verified using a handheld thermometer; duration, 15–20 min). The WSB procedure and hypothesized mechanism are illustrated in Figure 2. Patients in the control group did not receive medications currently known to promote stone expulsion. Nevertheless, all patients were advised to maintain a daily fluid intake of 2.0 L to facilitate stone passage. Oral diclofenac sodium 50 mg was allowed as rescue analgesia when stone-related pain became intolerable. During the treatment period, the total number of oral rescue analgesic doses taken and the daily frequency of renal colic episodes were recorded. Pain intensity was assessed using a 10-point visual analog scale, with 0 indicating no pain and 10 indicating the worst imaginable pain. Patients were instructed to record daily renal colic episodes and rescue analgesic use in a diary during the follow-up period. Pre-treatment VAS scores were recorded at enrollment, and post-treatment VAS scores were assessed at follow-up or after confirmed stone expulsion. Patients who suspected stone expulsion were asked to return to the hospital for appropriate evaluation to confirm the expulsion status. Participants were followed for up to 4 weeks unless confirmed stone expulsion occurred earlier or the patient withdrew consent. For patients who failed to pass the stone within 4 weeks, oral tamsulosin therapy or ureteroscopic stone removal was recommended.

Based on previous studies and preliminary clinical observations, we hypothesized that WSB would reduce the mean time to stone expulsion compared with conservative management alone. With a two-sided α level of 0.05, 80% power, and a 1:1 allocation ratio, the required sample size was estimated for comparison between two independent groups. After allowing for an estimated 10% dropout or loss-to-follow-up rate, the final sample size was set at 55 patients per group. The primary endpoint was stone expulsion time. Secondary endpoints included stone expulsion rates at weeks 1, 2, and 4, daily frequency of renal colic episodes, pre- and post-treatment VAS scores, rescue analgesic requirement, and adverse events. Continuous variables following a normal distribution were presented as mean ± standard deviation (SD) and compared using Student's *t*-test. Categorical variables were summarized as frequencies and compared using the chi-square test. Statistical analyses were performed using SPSS version 22.0 (IBM Corp., Armonk, NY, USA). A two-sided *P* value < 0.05 was considered statistically significant.

## Results

A total of 131 patients were assessed for eligibility between February 2023 and December 2023. After exclusion of 10 patients, 121 eligible patients were included in the final analysis, with 59 allocated to the WSB group and 62 to the control group. Baseline demographic and clinical characteristics were well balanced between the two groups, with no statistically significant differences in sex, age, BMI, stone size, laterality, hydronephrosis grade, or comorbidities (all *P* > 0.05; Table 1). Regarding the primary outcome, stone expulsion time was significantly shorter in the WSB group than in the control group (9.3 ± 2.9 days vs. 14.3 ± 4.2 days, *P* < 0.001). The WSB group demonstrated significantly higher stone expulsion rates (SER) at week 1 (40.7% vs. 21.0%, *P* = 0.019) and week 2 (81.4% vs. 61.3%, *P* = 0.015). No statistically significant difference was observed in the 4-week stone expulsion rate between the two groups (93.2% vs. 87.1%, *P* = 0.260; Table 2). With respect to pain-related outcomes, the WSB group experienced significantly fewer daily pain episodes (0.8 ± 0.3 vs. 1.3 ± 0.4, *P* < 0.001). Pre-treatment VAS scores were comparable between the two groups (5.9 ± 1.9 vs. 6.1 ± 2.0, *P* = 0.574), whereas post-treatment VAS scores were significantly lower in the WSB group (3.1 ± 1.0 vs. 4.2 ± 1.7, *P* < 0.001). The WSB group required significantly fewer rescue analgesics than the control group (4.1 ± 1.5 vs. 8.4 ± 3.1, *P* < 0.001). Regarding safety, no significant differences in adverse events were observed between the two groups, including dizziness, nausea, headache, palpitations, and constipation (all *P* > 0.05; Table 2).

**Table 1:** Baseline demographics and clinical characteristics of patients.

Variable	WSB (*N* = 59)	Control (*N* = 62)	*p*-value
Sex, *n*			
Male	32	40	0.250
Female	27	22	
Age (years)	42.4 ± 6.4	43.7 ± 5.9	0.248
BMI (kg/m^2^)	24.6 ± 5.2	24.3 ± 4.8	0.743
Stone size (mm)	3.8 ± 1.0	3.7 ± 1.2	0.619
Laterality, *n*			
Left	29	33	0.654
Right	30	29	
Hydronephrosis, *n*			
Negative or mild	49	55	0.371
Moderate	10	7	
Hypertension, *n*			
Yes	20	20	0.848
No	39	42	
Diabetes, *n*			
Yes	15	19	0.523
No	44	43	
Smoke, *n*			
Yes	24	29	0.499
No	35	33	

Data are presented as mean ± SD or *n* (%). BMI, body mass index.

**Table 2:** Comparison of clinical outcomes between two groups.

Outcome	WSB (*N* = 59)	Control (*N* = 62)	*p*-value
Stone expulsion rate (SER), *n* (%)			
1st-week follow-up	24 (40.7%)	13 (21.0%)	0.019^*^
2nd-week follow-up	48 (81.4%)	38 (61.3%)	0.015^*^
4th-week follow-up	55 (93.2%)	54 (87.1%)	0.260
Stone expulsion time (days)	9.3 ±2.9	14.3 ± 4.2	< 0.001^***^
Pain episodes (per day)	0.8 ± 0.3	1.3 ± 0.4	< 0.001^***^
Visual analog scale (VAS)			
Before treatment	5.9 ± 1.9	6.1 ± 2.0	0.574
After treatment	3.1 ± 1.0	4.2 ± 1.7	< 0.001^***^
Analgesic requirement (times)	4.1 ± 1.5	8.4 ± 3.1	< 0.001^***^
Adverse events, *n*			
Dizziness	4	3	0.713
Nausea	4	3	0.713
Headache	5	4	0.739
Palpitations	5	1	0.108
Constipation	2	1	0.613

SER, stone expulsion rate; ^*^P < 0.05; ^***^P < 0.001.

## Discussion

For DUC ≤5 mm, current evidence suggests that spontaneous passage rates are already high (5, 11). A systematic review by Pearce et al. reported an overall spontaneous passage rate of 87% for distal ureteric calculi ≤5 mm, with expulsion times ranging from 8.54 to 24.5 days (11). In addition, Miller and Kane reported that 95% of ureteral stones (2–4 mm) passed spontaneously, although passage could take as long as 40 days (16). Meanwhile, the benefit of MET appears limited in small stones, which partly explains why observation remains the preferred strategy in many patients with DUC < 5 mm (5–10). WSB is a simple local heat-based intervention in which the perineal and gluteal regions are immersed in warm water for a defined period in order to relieve pain, reduce muscle spasm, and improve local comfort. As noted above, WSB has not previously been investigated in the context of ureteral stone management. In our study, patients in the WSB group were instructed to perform a daily WSB, and the great majority completed the intervention at home. The significantly higher SERs at weeks 1 and 2, but not at week 4, suggests that WSB primarily accelerates the early phase of stone passage rather than altering the ultimate expulsion outcome. The mechanism by which WSB may facilitate earlier passage of small DUC remains speculative, but several pathophysiological considerations support this possibility. Ureteral obstruction caused by stones can increase intraluminal pressure and promote prostaglandin-mediated ureteral spasm and ureteral wall edema, both of which may impede stone passage (17–19). As a local heat-based intervention, WSB may help relieve smooth muscle spasm, reduce pain-related muscular tension, and improve patient tolerance during expectant management, thereby potentially reducing resistance to stone movement along the ureter. This interpretation is also supported indirectly by physiological studies of thermal stimulation. Cui et al. (20) showed that repeated warm water baths reduced resting sympathetic activity and heart rate in humans, suggesting that thermal stimulation may attenuate sympathetic activation and pain-related stress responses. Shafik et al. (21) proposed a possible “thermo-sphincter reflex,” suggesting that warm water stimulation may induce reflex relaxation of the internal urethral sphincter. Although that study concerned urethral sphincter relaxation rather than the ureter, it nevertheless suggests that warm water exposure may influence urinary tract smooth muscle through reflex mechanisms (21).

Beyond its potential effect on stone passage, another clinically relevant finding in our study was that the WSB group experienced significantly fewer daily pain episodes (0.8 ± 0.3 vs. 1.3 ± 0.4, *P* < 0.001), lower post-treatment VAS scores (3.1 ± 1.0 vs. 4.2±1.7, *P* < 0.001), and lower analgesic requirements (4.1 ± 1.5 vs. 8.4 ± 3.1, *P* < 0.001) than the control group. Such an effect may be valuable because it not only improves patient comfort but may also reduce reliance on repeated analgesic use during the waiting period. These findings are broadly consistent with previous studies evaluating local heat therapy for renal colic. Kober et al. (22) demonstrated in a randomized trial that local active warming significantly reduced pain, anxiety, and nausea in patients with acute renal colic. Similarly, Mutlu et al. (23) reported that local heat-patch therapy significantly lowered pain scores and reduced the need for rescue analgesics in patients with urolithiasis. However, both of those studies evaluated local heat application during acute pain episodes, whereas our study examined scheduled daily warm sitz baths throughout the observation period. Therefore, our findings suggest that WSB may have potential value not only for acute pain relief but also for symptom control during the conservative management of small DUC. Taken together, these observations provide indirect support for the hypothesis that WSB may improve conditions favorable to stone passage, particularly in patients with small DUC.

WSB also has several advantages in terms of real-world applicability. The intervention can be performed at home and is easy to implement. Moreover, in many countries, soaking in a bathtub or using warm water immersion is already a familiar daily habit, which may facilitate broader adoption of this strategy in appropriately selected patients. In our study, no significant difference in adverse events was observed between the two groups, indicating that WSB did not increase adverse events and may be regarded as a safe intervention for DUC ≤5 mm. These findings should be interpreted within the context of distal ureteral calculi ≤5 mm. The results may not be generalizable to larger stones, proximal or mid-ureteral stones, impacted stones, or patients requiring urgent intervention.

However, this study has several limitations. First, this was a single-center study with a relatively small sample size, and larger multicenter studies are needed to further validate the therapeutic value of WSB for DUC. Second, the WSB was performed by patients at home. Although standardized instructions were provided, complete uniformity in water temperature, duration, and adherence was difficult to ensure, which may have introduced variability in the results. Third, our study focused exclusively on DUC ≤5 mm. Further studies are warranted to explore whether WSB may also be beneficial for larger stones or stones located in other parts of the ureter. Fourth, different imaging modalities were used for stone evaluation, which may have introduced measurement discrepancies. Although ultrasonography was used as the first-line follow-up modality to minimize radiation exposure, the sensitivity of different imaging methods for small ureteral stones may vary. Finally, because the WSB intervention could not be blinded, subjective outcomes such as pain intensity and analgesic use may have been influenced by expectation and attention effects. Therefore, although the reductions in pain episodes, VAS scores, and rescue analgesic use are clinically meaningful, these findings should be interpreted with caution.

## Conclusion

WSB represents a simple, safe, and non-pharmacological adjunctive intervention for patients with DUC ≤5 mm. Daily WSB may accelerate early stone passage and reduce pain burden without increasing adverse events; however, it did not significantly improve the final 4-week SER. Given its low cost and ease of home application, WSB may be considered a practical supportive measure during expectant management of DUC ≤5 mm.

## Data Availability

The raw data supporting the conclusions of this article will be made available by the authors, without undue reservation.
